# Therapeutic Impact of Aerobic Exercise on Adolescents with Obesity and Its Association with Expression of miRNAs and Cytokines: A Clinical Approach

**DOI:** 10.3390/medicina60030459

**Published:** 2024-03-10

**Authors:** Petricia Hillari Raj, Prasanth Subramanian, Mohanraj Nehru, Saravanan Ayyavoo, Nachal Annamalai, Venkataraman Prabhu

**Affiliations:** 1 Department of Physiology, Trichy SRM Medical College Hospital & Research Centre, Irungalur, Trichy 621105, Tamil Nadu, India; hillari06@gmail.com (P.H.R.); nachal.a@gmail.com (N.A.); 2 Department of Medical Research, SRM Medical College Hospital & Research Centre, Kattankulathur, Chennai 603203, Tamil Nadu, India; ps0546@srmist.edu.in (P.S.); mn4147@srmist.edu.in (M.N.); 3 Department of Physiology, SRM Medical College Hospital & Research Centre, Kattankulathur, Chennai 603203, Tamil Nadu, India; drsaravanan1977@gmail.com

**Keywords:** obesity, biomarkers, circulating miRNAs, aerobic exercise

## Abstract

*Background and Objectives*: MicroRNAs are short noncoding RNAs that play an essential role in controlling gene expression at the posttranscriptional level. They can serve as biomarkers in the management of obesity. Circulating miRNAs levels change with exercise, impacting various physiological and biological systems, including structural and functional changes. *Aim*: The purpose of this study is to evaluate the levels of miRNAs 423-5p and 128-1 in young adolescents with obesity before and after an aerobic exercise programme. We also analyse the relationship between those microRNAs and obesity-related parameters in response to aerobic exercise training. *Materials and Methods*: A total of 64 adolescent individuals (32 individuals with obesity and 32 healthy individuals) were enrolled in the study to participate in a 6-month aerobic exercise programme. Anthropometric measurements, biochemical parameters and blood samples were collected from all the participants prior to exercise training and after the 6-month programme. Gene expression analysis of the study participants was performed using quantitative real-time PCR. *Results*: Expression levels of circulating microRNAs 423-5p (*p* < 0.01) and 128-1 (*p* < 0.01) differed significantly before and after exercise in the study population. Circulating miRNA 423-5p increased and correlated significantly with BMI while circulating miRNA 128-1 decreased and also significantly correlated with BMI after the 6-month aerobic exercise programme. Logistic regression analysis shows that the elevation in miRNAs expression levels has a strong significant association with the increased levels of the cytokines IL-6 and TNF-α (*p* < 0.05). *Conclusions*: Obesity leads to alterations in the expressions of miRNA 423-5p and miRNA 128-1. The significant changes observed after an aerobic exercise programme demonstrate the potential of these miRNAs as diagnostic and prognostic biomarkers for obesity.

## 1. Introduction

Obesity is one of the world’s most pressing health problems, responsible for 5% of total global mortality. It is a chronic disease, characterised by the presence of excessive body fat and an increase in the size and number of adipocytes in the body [1]. The World Health Organization (WHO) and other international organizations like the American Medical Association officially recognise obesity as a global health disease [1,2]. The Indian National Family Health Survey -5 (2019–2021) indicates a concerning rise in obesity prevalence among Indian adults in the 15–49 age group. Obesity rates increased from 21% to 24% in women and 19% to 23% in men [3]. The prevalence of being overweight and having obesity among Indian youth has also escalated. According to the ICMR-INDIAB-3 survey, (covering three states and one union territory), the prevalence of being overweight/having obesity in rural Tamil Nadu was 29.9% [4]. Obesity remarkably affects quality of life and reduces longevity [5]. Projections suggest that by 2025, 18% of men and 21% of women worldwide will have obesity. Obesity and its problems cost the global economy USD 2 trillion each year. This financial burden could be equivalent to 2.8% of global gross domestic product (GDP) [6]. The International Obesity Task Force (IOTF) and the World Health Organization (WHO) have declared obesity a 21st-century epidemic [7]. In developing countries like India, a confluence of factors contributes to rising obesity rates and increased body mass index (BMI). These include urbanisation, increased reliance on mechanised transport, the proliferation of fast foods, decreased physical activity and excessive television viewing time [8]. While genetic factors and epigenetic predisposition play a role, external influences are considered crucial in the development of obesity [2]. Research into gene expression mechanisms during obesity development offers valuable insights for prevention, early diagnosis and effective management [9].

MicroRNAs (miRNAs) are endogenously initiated, short noncoding single-stranded, 21–23 nucleotides long RNAs that negatively regulate or repress target gene expression at the posttranscriptional level by pairing with the 3′-untranslated region (UTR) of their target mRNA. These miRNAs are expressed in all cell types. They play an important role in both normal physiological processes and in the development of diseases such as obesity and cancer [2,9].

In addition to tissues, these miRNAs are also found in body fluids such as serum, plasma, saliva, sweat, cerebrospinal fluid, tears, breast milk, peritoneal fluid, bronchial lavage, seminal fluid, follicular fluid and urine [10,11]. These circulating microRNAs, unlike regular miRNAs, are highly stable when subjected to extreme conditions like boiling, high pH, prolonged storage time and multiple freeze–thaw cycles, while most cellular RNAs are rapidly destroyed [10,11]. Circulating miRNAs are protected from degradation by endogenous RNAase activity because they are contained in small membranous vesicles e.g., exosomes, exosome-like vesicles, microparticles and apoptotic bodies, packed with high-density lipoproteins (HDLs) and linked to RNA binding proteins. As they are highly stable and easily detectable, they are very good targets for extraction and quantification [11,12].

Aerobic exercises are chosen as a management tool for obesity. Aerobic exercise is any physical activity that will increase oxygen consumption. It is treated as the most effective exercise to lower body weight and BMI [8]. The American College of Sports and Medicine defines aerobic exercise as “any activity that uses large group of muscles, can be maintained continuously, and is rhythmic in nature”. Walking, cycling, jogging, running and swimming are all examples of aerobic exercise [8]. Through cloning, sequencing and quantification, in 2008, Patrick and associates demonstrated that human plasma contains endogenous miRNAs with a length of 18 to 24 nucleotides called circulating miRNA or extracellular miRNA, which are present in various biological fluids [13,14]. Some circulating miRNAs are specifically associated with obesity, for instance, circulating miRNA 423-5p [12,15,16] and circulating miRNA-128-1 [17]. Understanding how exercise affects gene regulation at the level of miRNAs will be crucial in optimising the therapeutic benefits of aerobic exercise for human health [18].

miRNAs have become important metabolic process mediators, maintaining or changing physiological processes like metabolic homeostasis and energy balance. In investigations on both humans and animals, altered miRNA expression has been linked to obesity. Adipose tissue, the pancreas, the liver, and muscles are just a few of the tissues and organs whose status and activities may be impacted by the dysregulation of miRNAs. This could potentially contribute to metabolic abnormalities and diseases linked to obesity [19]. During adipogenesis, miRNAs regulate the release and function of signals (adipokines, inflammatory agents and exosomes) from adipose tissue. All of the above findings demonstrate why there is an interest in studying the function of miRNAs in obesity and related disorders. A shift in miRNA expression may cause changes in the pattern of genes controlling a number of biological processes, such as adipogenesis, adipose tissue inflammation, lipid metabolism and insulin resistance [20]. Despite researchers’ efforts to uncover the link between obesity and miRNAs, the importance of circulating miRNAs for obesity remains unknown. With regard to obesity, different studies have revealed different miRNA expression profiles. Many studies have concentrated on animal systems, so there is a paucity of data on human individuals. When compared to the Western population, Asian populations have a characteristically lower body mass index and a higher percentage of fat at a given BMI. These factors should be considered for a full understanding of obesity-related miRNAs [21].

The aim of the study was to analyse the circulating miRNA 423-5p and miRNA 128-1 levels and their association with cytokines (TNF-α and IL-6) before intervention with adults with obesity and lean adults of the 18–23 age group. The study also evaluates the effect of exercise training on miRNA 423-5p and miRNA-128-1 and the blood parameters in adults with obesity.

## 2. Methodology

### 2.1. Ethical Approval and Informed Consent

Ethical approval from the institutional ethical committee of TSRMMCH and RC was obtained to conduct the study (14/TSRMMCH and RC/ME-1/2020-IEC N0:20, dated on 31 January 2020). The aim and procedure of the study were explained to the subjects and written consent was obtained from each subject. After taking a detailed medical history, anthropometric measurements and thorough physical examinations were performed. All the parameters were measured before and after the 6-month aerobic exercise programme.

### 2.2. Anthropometric Measurements

BMI = weight (kg)/height (m)^2^. The maximum limit of normal BMI according to WHO guidelines is 24.9 kg/m^2^. Therefore, subjects in this study were divided into two groups: one with a BMI < 25 and the other with a BMI ≥ 25. After an overnight fast, the waist circumference was measured using a tape measure. To reduce abdominal tension, the participants were instructed to relax and take a few deep breaths before the measurement. Subjects were asked to stand with their feet together, arms at their sides and weight evenly distributed across their bare feet. In a standing position, the waist circumference was measured at the midpoint between the iliac crest and the lowest point of the rib cage at the end of normal breathing. The hip circumference was measured at the level parallel to the floor around the largest circumference of the buttocks [22].

### 2.3. Blood Pressure Measurement

Blood pressure was measured using a mercury sphygmomanometer. Participants were instructed to relax while remaining seated for five minutes. Two readings were taken every minute. If the difference between the first two readings exceeded 5 mm Hg, a third reading was taken and the average of all three values was used [23,24].

### 2.4. Pulse Rate

The participant was directed to assume a comfortable posture. The pulse rate was measured for one minute at the wrist while their forearm was held in a partially rotated position with the wrist slightly bent [25].

### 2.5. Sample Collection and RNA and DNA Extraction

According to the specified protocol, blood samples were collected and preserved from the participants in the morning. The samples were then centrifuged. The resulting serum and whole blood were transported in ice storage boxes to the laboratory for immediate extraction. RNA was extracted from the serum samples using the PureFast^®^ miRNA mini spin purification kit (HELINI Biomolecules, Chennai, India). DNA was extracted from the whole blood using the Qiagen QIAamp DNA Mini Kit (Qiagen, Hilden, Germany). The purity and concentrations of DNA were determined spectrophotometrically. Only samples with an OD260/OD280 ratio greater than 1.8 were used for gene expression studies.

### 2.6. Gene Expression Analysis

Real-time quantitative PCR was performed to analyse gene expression. miRNA and cytokines were amplified in the presence of gene-specific primers and the housekeeping gene GAPDH. The primer concentration was 20 pmol for GAPDH and gene-specific primers. PCR amplification was performed using a Bio-Rad CFX384 Touch Real-Time PCR (Bio-Rad, Hercules, CA, USA) under the following cycle conditions: 45 cycles of denaturation at 95 °C for 15 s, annealing/extension at 60 °C for 1 min and an initial cycle of 50 °C for 2 min. GAPDH was used to normalise the relative quantification of the corresponding gene expression levels, which was computed using the 2^−ΔΔCt^ method. The target genes 423-5p and 128-1 miRNAs and the cytokines (IL-6 and TNF-α) were quantified using qPCR. The primers were listed in Table 1.

### 2.7. Aerobic Exercise Programme

Subjects were asked to perform a moderate-intensity aerobic exercise programme for 150 min per week for 6 months [26]. They were asked to do brisk walking (4 km/h MET score-3–6) and complete 10,000 steps/day calculated by a step tracker. Instructions were provided so that the participants were to increase their walking distance by 1000 steps each week, to reach at least 10,000 steps per day [27,28]. Their heart rate (HR) was also monitored before and after aerobic training sessions. The American College of Sports Medicine guidelines define moderate-intensity exercise as having a maximum HR between 64% and 76% of a person’s capacity. Each training session consisted of a 15-min warm-up, followed by 30 min of exercise for a total of 45 min. The session concluded with 10 min of stretching exercises performed five days a week. In addition to aerobic training, subjects also performed strengthening exercises for individual muscles.

### 2.8. Statistical Analysis

Data were analysed using SPSS (Statistical Package for the Social Sciences) software version 21. Descriptive statistics were used: Continuous or numerical variables such as age were represented by mean and standard deviation. ANOVA was used to test the difference in the means between three or more groups. The paired-samples ‘*t*’ test was used to test for differences between the two groups. When a continuous variable was associated with another continuous variable, Pearson’s correlation test was used after confirming normality. Linear regression was used to determine predictors for the outcome variable. *p*-values less than 0.05 were considered statistically significant.

## 3. Results

The demographic characteristics of the study population are shown in Table 2. The study included two groups: an intervention group with 32 subjects with obesity (50%) and a control group with 32 subjects without obesity (50%) who did not receive the intervention. There was an equal distribution of males (*n* = 16) and females (*n* = 16) in both groups. The groups were similar in terms of age, sex, heart rate, diastolic blood pressure, fasting blood glucose and lipid levels including triglycerides, VLDL-cholesterol and HDL–cholesterol. However, BMI, waist circumference, hip circumference, waist/hip ratio, systolic blood pressure, total cholesterol, LDL-cholesterol and C-reactive protein were significantly higher in subjects with obesity.

Table 2 expresses increased levels of BMI, waist circumference (cm), hip circumference (cm), waist/hip ratio (WHR), FBS, PPBS, total cholesterol and HbA1c between the control subjects and subjects with obesity. After the intervention, the test group (subjects with obesity) experienced a significant reduction in weight, BMI, waist circumference (cm), hip circumference (cm), waist/hip ratio (WHR), FBS, PPBS, total cholesterol and HbA1c (*p* < 0.05) as measured during the pretest and posttest of the aerobic exercise programme. The levels between the healthy control and test groups also had a significant difference.

**Figure 1:**
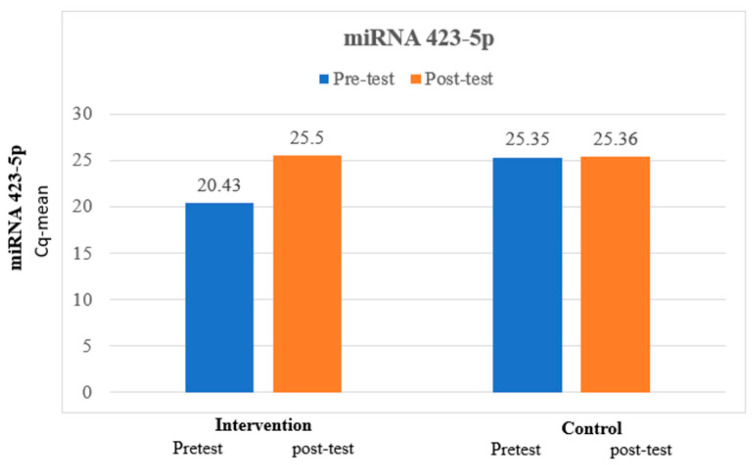
Comparison of miRNA 423-5p between intervention and control group.

The mean miRNA 423-5p at pretest was 20.43, which is lower than the mean miRNA 423-5p at posttest, which was 25.5, and the difference between miRNA 423-5p at pretest and miRNA 423-5p at posttest was statistically significant among the intervention group. The mean miRNA 423-5p at pretest was 25.35, which is lower than the mean miRNA 423-5p at posttest, which was 25.36, and the difference between miRNA 423-5p at pretest and miRNA 423-5p at posttest was not statistically significant among the control group. The paired *t*-test for comparison of the analysis is exhibited in Table 3 and expressed in Figure 1.

**Figure 2:**
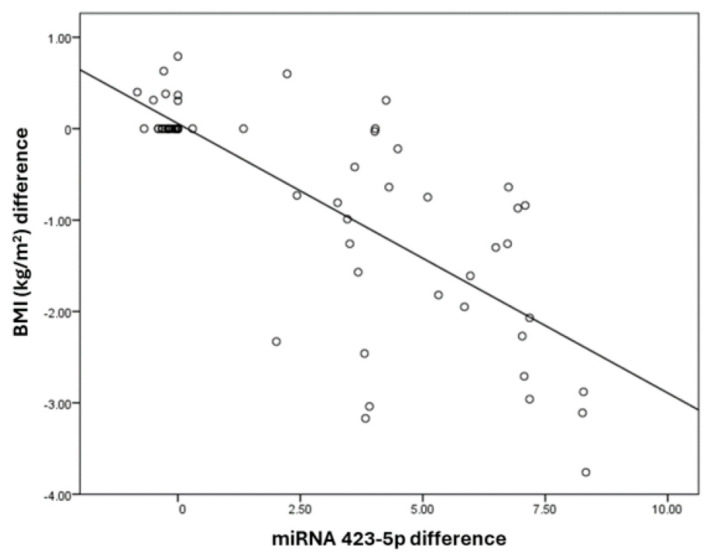
Correlation between miRNA 423-5p and BMI (kg/m^2^) differences.

The miRNA 423-5p difference has a negative correlation with BMI (kg/m^2^), a difference with a correlation coefficient of −0.54. BMI (kg/m^2^) and the difference decreases by −0.3 times for each unit increase in miRNA 423-5p difference. The correlation between the BMI (kg/m^2^) difference and miRNA 423-5p difference was statistically significant (Table 4). The correlation between miRNA 423-5p with BMI is exhibited in Figure 2.

**Figure 3:**
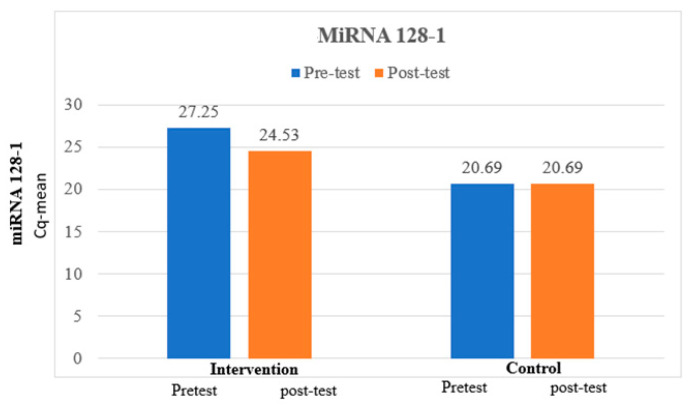
Comparison of miRNA 128-1 between intervention and control groups.

The mean miRNA 128-1 at pretest was 27.25, which is higher than the mean miRNA 128-1 at posttest, which was 24., and the difference between miRNA 128-1 at pretest and miRNA 128-1 at posttest was statistically significant among the intervention group. The mean miRNA 128-1 at pretest was 20.69, which is similar to the mean miRNA 128-1 at posttest, which was 20.69, and the difference between miRNA 128-1 at pretest and miRNA 128-1 at posttest was not statistically significant among the control group, which is expressed in Table 5 and Figure 3.

**Figure 4:**
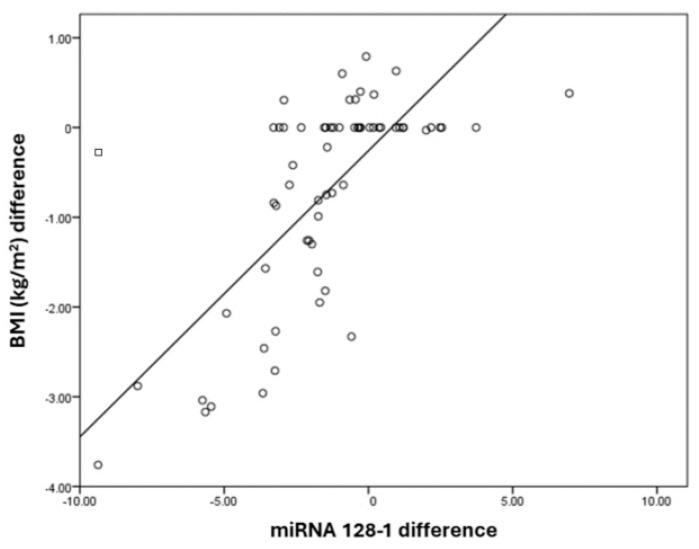
Correlation between miRNA128-1 and BMI (kg/m^2^) differences.

The miRNA128-1 difference has a positive correlation with the BMI (kg/m^2^) difference, with a correlation coefficient of 0.79. BMI (kg/m^2^) and the difference increases by 0.39 times for each unit increase in the miRNA128-1 difference. The correlation between the BMI (kg/m^2^) difference and miRNA128-1 difference was statistically significant (Table 6). It is represented in the Figure 4.

**Figure 5:**
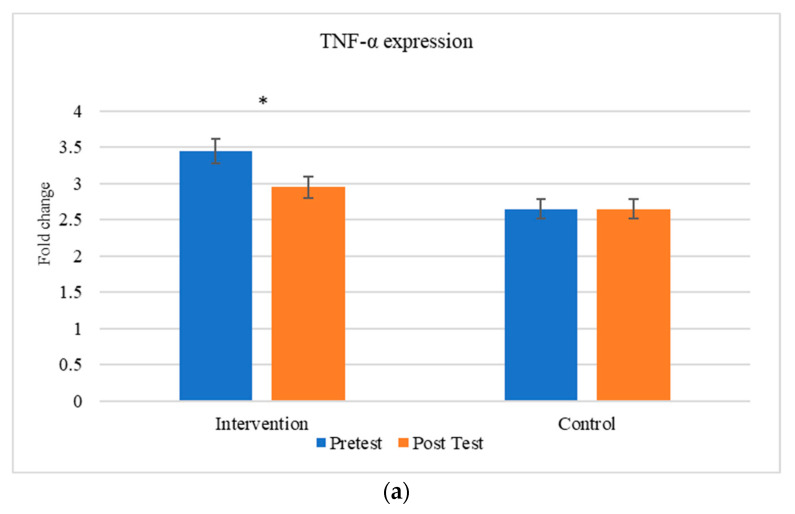

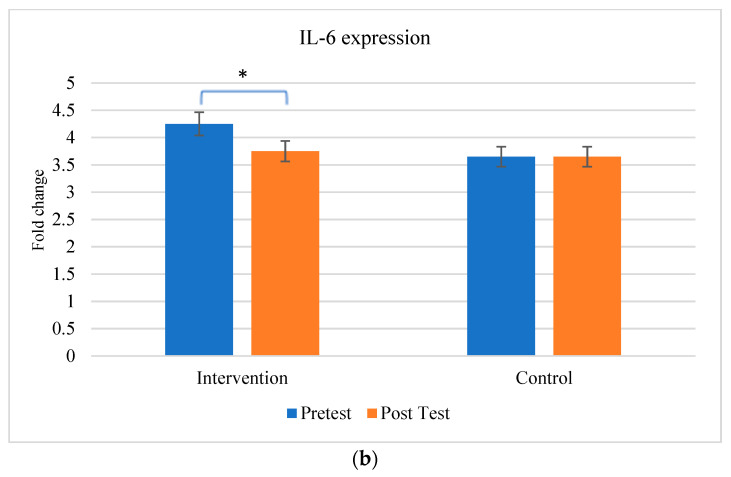
Comparison of inflammatory markers between intervention and control groups (**a**) TNF-α and (**b**) IL-6. * The results are statistically significant at *p* < 0.05.

The comparison between the gene expression of cytokines (TNF-α and IL-6) were exhibited in Figure 5. There is a significant change in the expression of cytokines between the individuals before and after the aerobic exercise training.

### Association between the Expression of Cytokines and miRNA Genes

**Figure 6:**
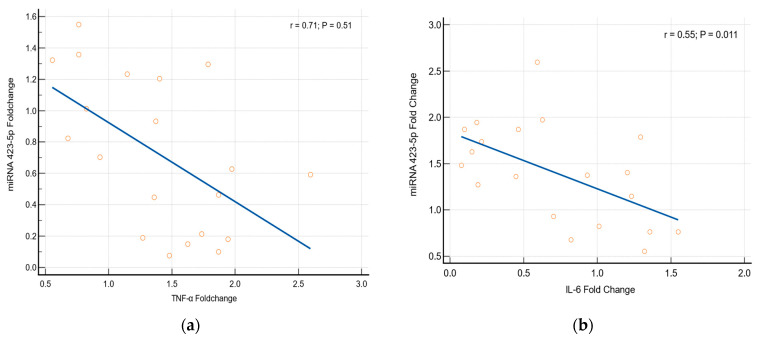

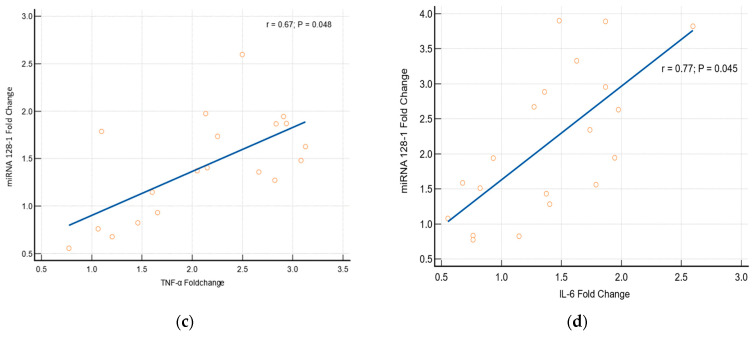
Association between cytokines and miRNAs (**a**) TNF-α with miRNA 423-5p, (**b**) IL-6 with miRNA 423-5p, (**c**) TNF-α with miRNA 128-1 and (**d**) IL-6 with miRNA 128-1.

miRNAs 423-5p and 128-1 are usually stimulated by the inflammatory responses. Our study results found that there is a significant alteration in the expression of miRNAs 423-5p and 128-1 genes related to cytokines (TNF-α and IL-6). Logistic regression statistics prove that the alteration in the expression of miRNAs genes has a strong significant association with an elevation of the cytokines [miRNA-423-5p with TNF-α (*p* = 0.051, r = 0.71) and miRNA-423-5p with IL-6 (*p* = 0.011, r = 0.55); miRNA-128-1 with TNF-α (*p* = 0.048, r = 0.67) and miRNA 128-1 with IL-6 (*p* = 0.045, r = 0.77)], which is depicted in Figure 6.

## 4. Discussion

The purpose of the study was to analyse the miRNAs 423-5p and miRNA 128-1 levels before intervention with lean adults and adults with obesity and to evaluate the effect of exercise training on miRNA 423-5p and miRNA -128-1 in adults with obesity. The current study shows expression levels of circulating microRNAs 423-5p and 128-1 significantly differed before and after exercise in the study population (*p* < 0.001). Circulating miRNA 423-5p increased and correlated significantly with BMI while circulating miRNA 128-1 decreased and correlated significantly with BMI after a 6-month aerobic exercise programme. 

In a study by Al-Rawaf (2019), at least 10 circulating miRNAs, including elevated levels of miR-142-3p, miR-140-5p, miR-222, miR-143, and miR-130 and decreased levels of miR532-5p, miR-423-5p, miR-520c-3p, miR-146a, and miR-15a, were found in overweight adolescents and adolescents with obesity. These miRNAs showed a strong correlation with various obesity markers, including body mass index (BMI), waist/hip ratio (WHtR), adipokines, adiponectin, leptin and fasting blood sugar levels (FBS), insulin, HOMA-IR, triglycerides (TG), HDL-C, C-peptide and LDL-cholesterol [15]. Prepubertal obesity further demonstrated significant deregulation of 15 distinct circulating miRNAs. This included lower levels of miR-221 and miR-28-3p and elevated plasma concentrations of miR-486-5p, miR-486-3p, miR-142-3p, miR-130b and miR-423-5p (*p* < 0.001) [29]. These miRNA concentrations correlated significantly with body mass index including percent fat mass, waist and regional fat distribution and other obesity measures as well as laboratory parameters like adiponectin, C-reactive protein and lipids [29]. Compared to the control group, males with obesity and morbid obesity exhibited differences in 18 circulating miRNAs (*p* < 0.05). Notably, individuals with morbid obesity showed an increased expression of miR-142-3p, miR-140-5p and miR-222 along with lower levels of miR-221, miR-15a, miR-520c-3p, miR-423-5p and miR-130b. These miRNA (*p* < 0.001) levels strongly correlated with BMI and other measures of obesity [16]. Recent research has identified miRNA 128-1 as a regulator of energy expenditure, influencing obesity and glucose metabolism [30]. 

According to studies, miR-128-1 is crucial for maintaining the balance of lipid and energy levels. MiR-128-1 is continuously inhibited in hyperlipidaemic Apoe/mice, resulting in a significant reduction in hepatic steatosis, VLDL-associated TAGs and circulating VLDL-C/LDL-C. By specifically targeting the 3′UTR of low-density lipoprotein receptors (LDLR) and the ATP-binding cassette A1 (ABCA1), miR-128-1 regulates the circulating lipoprotein metabolism. Additionally, miR-128-1 inhibition increases hepatic insulin sensitivity, which improves glucose clearance. Furthermore, miR-128-1 also controls ABCA-1 expression in macrophages. When miR-128-1 regulates, ABCA1 expression and macrophage cholesterol efflux are both increased. These results suggest miR-148 and miR-128-1 inhibition could be potential therapeutic strategies for treating dyslipidemia, obesity and CVD [31]. However, obesity is a heritable trait in humans. The mir-128-1 gene is situated at the human 2q21.3 locus, which has syntenic loci in dogs and cattle that are associated with these animals. Adipose tissue, muscle and the liver are three tissues where miR-128-1 is widely expressed. In these tissues, miR-128-1 regulates the homeostasis of circulating lipoproteins. Along with other regulators of fatty acid oxidation, mitochondrial energy expenditure and inflammation, it also controls the expression of genes encoding PPAR transcription factors, which are involved in metabolic balance, lipid, glucose, and energy metabolism, adipogenesis and inflammation. PPAR transcription factors are a group of nuclear receptor proteins that regulate the activity of several genes involved in this process. Free fatty acids, eicosanoids and vitamin B3 are all endogenous ligands for peroxisome proliferator-activated receptors (PPARs). Overall, miR-128-1 inhibits gene expression controlling energy expenditure in various metabolically active organs and tissues. Loss of miR-128-1 function (through inhibition) consequently lowered weight gain and fat deposition in mice fed a calorie-rich diet. This effect was connected with improved insulin sensitivity. Therefore, people with the 2q21.3 variation (e.g., a specific genetic variant) have higher levels of miR-128-1 expression and more accessible chromatin at the mir-128-1 gene. This increased expression of miR-128-1 may predispose them to obesity, making this miRNA a promising target for the treatment of obesity [32].

There is an increasing body of literature that highlights the potential of miRNAs as valuable clinical instruments, specifically in the context of identifying “circulating” miRNAs as biomarkers. Optimal blood glucose levels are maintained through the precise regulation of insulin release. Recently recognised as “ribo-regulators” of glucose homeostasis, microRNAs (miRNAs) influence the sensitivity or resistance of the target tissues while playing a crucial role in the production and secretion of insulin [33,34,35,36], influenced by various factors like metabolism, caloric and food consumption and lack of physical activity. Omics methodologies suggest a link between obesity or metabolic diseases and the expression of multiple microRNAs in various tissues (e.g., adipose tissue, liver and pancreas). Additionally, as supported by several studies, the expression of miRNAs was found to be directly correlated with diet and lifestyle. Several studies indicate that the miR-17/20/93 family, miR-21/590-5p family, miR-200b/c family, miR-221/222 family, let-7/miR-98 family and miR-203 are the most dysregulated in this context, despite the importance of the list of miRNAs associated with diet [20]. The outcomes of our research are consistent with the theoretical and empirical findings mentioned earlier. The gene expression levels of the circulating 128-1 and 423-5p microRNAs were significantly altered in adolescents with obesity relative to healthy individuals. Additionally, individuals engaging in aerobic exercise training for six months exhibited beneficial outcomes with a reduction in miRNA expression observed in the exercise group. The results of this study suggest that circulating microRNAs with the sequences 423-5p and 128-1 might possess diagnostic potential in relation to obesity.

Logistic regression analysis shows that the elevated expression of miRNA-128-1p has a strong significant association with the elevation of the cytokines (TNF-α and IL-6). Zhang et al.’s results express that understanding the clinical significance of serum miRNA-128 is crucial for improving diagnosis and treatment. Additionally, their correlation analysis indicated positive correlations between serum miR-128 levels and serum IL-1β and the TNF-α levels in neurological disorder patients [37]. Arcidiacono et al.’s research indicates that proinflammatory factors, including CRP, and cytokines, like IL-6, TNF-α and INF-γ, contribute to insulin resistance in individuals with obesity [38]. Combining the findings from Zhang et al., Arcidiacono et al. [37,38] and our research suggests a strong positive correlation between the miRNA-128 family and cytokines, suggesting miRNA-128 to be a potential diagnostic marker in diseased conditions.

The effective application of logistic regression statistics reveals a robust and significant correlation between the upregulation of miRNA-423-5p and the increase in cytokines (TNF-α and IL-6). Yang et al.’s research demonstrates that miRNA-423-5p expression significantly increased in an inflammatory environment and decreased in a treated animal model, revealing an indirect association between the levels of IL-6 in disease states [39]. Ibarra et al.’s review highlights a direct correlation between miRNA expression and inflammation and obesity, proposing these molecules as potential therapeutic targets [40]. Integrating previous research, review findings and our study results, we find a robust association between miRNA-423-5p and cytokines, suggesting its potential as a promising diagnostic and therapeutic indicator in pathological states.

## 5. Conclusions and Future Prospects

Substantial progress has been made in understanding how microRNAs function and their potential as biomarkers for various diseases. The current research discovered that obesity alters the expressions of miRNA 423-5p and miRNA -128-1. Additionally, significant changes related to the aerobic exercise programme suggest these miRNAs can serve as both diagnostic and target predictive biomarkers for obesity. Knowledge gained regarding miRNAs is expected to be extensively applied in medicine in the coming years. Despite ongoing challenges, miRNAs hold undeniable potential in prognosis, diagnosis and targeted therapy development.

## Figures and Tables

**Table 1:** Forward and reverse primer sequences for qRT-PCR.

Gene	Forward Primer (5′ to 3′)	Reverse Primer (3′ to 5′)
**GAPDH** **(House Keeping)**	ATGGGGAAGGTGAAGGTCG	TAAAAGCAGCCCTGGTGACC
**miRNA 423-5p**	GCTATCAAGCTCCATCCGCAT	TAAGACGAAGCACCGGA
**miRNA 128-1**	CCGCCGGGATCCGCAGAAAGTCAAC	CGCCGAAGCTTATCC
**TNF-α**	TGTTCTGGAGGTACTCTAGG	TGTTCTGGAGGTACTCTAGG
**IL-6**	ATCATTCTCTAGTGTCTGGTTGG	TGTTCTGGAGGTACTCTAGG

**Table 2:** Demographic and metabolic characteristics of study participants.

	Group			ANOVA *p* Value
	Controls (*n* = 32)	Pretest Obesity (*n* = 32)	Posttest Obesity (*n* = 32)	
**Age (years)**	18.8 (±1)	18.4 (±0.7)		
**Height (cm)**	166.8 (±9.5)	165.7 (±11.1)		
**Weight (kg)**	61.9 (±9.2)	78.7 (±16.3)	74.3 (±16.1)	0.001
**BMI (kg/m^2^)**	22.1 (±1.7)	28.4 (±3.1)	26.7 (±3.3)	0.001
**Waist Circumference (cm)**	75.5 (±10.4)	90.7 (±11.4)	88.4 (±12.7)	0.001
**Hip Circumference (cm)**	96.8 (±5.9)	106.9 (±9.6)	104.6 (±7.8)	0.001
**Waist/Hip Ratio**	0.7 (±0.1)	0.85 (±0.07)	0.84 (±0.1)	0.001
**SBP (mmHg)**	108.3 (±10.5)	119.7 (±13.9)	116.6 (±14.5)	0.002
**DBP (mmHg)**	75.6 (±14)	75.3 (±9.4)	73.1 (±9.5)	0.658
**Heart Rate (bpm)**	80.9 (±6.2)	84.8 (±9.3)	83.7 (±9.6)	0.179
**Fasting Blood Sugar (mg/dL)**	75.1 (±8.2)	81.8 (±44.6)	78.4 (±35.4)	0.72
**Post Prandial Blood Sugar (md/dL)**	123 (±6.2)	176 (±18.3)	164 (±21.5)	0.048
**HbA1c**	5.54 (±0.2)	6.52 (±1.3)	6.36 (±2.3)	0.05
**Total Cholesterol (mg/dL)**	121.9 (±23.2)	146.3 (±18.5)	141.1 (±20.5)	0.001
**Triglycerides (mg/dL)**	81.5 (±21.5)	75.8 (±17.8)	64.8 (±15.4)	0.812
**HDL-Cholesterol (mg/dL)**	50.8 (±9.9)	43.1 (±7.8)	49.1 (±6.6)	0.002
**LDL-Cholesterol (mg/dL)**	88.3 (±12.9)	88 (±15.2)	85.8 (±17.1)	0.765
**VLDL-Cholesterol (mg/dL)**	15.4 (±4.5)	15.1 (±8.1)	12.9 (±4.7)	0.194
**CRP (mg/L)**	0.8 (±0.7)	3.6 (±4.6)	3.2 (±3.4)	0.002

SBP: systolic blood pressure, DBP: diastolic blood pressure, HDL: high-density lipoprotein, LDL: low-density lipoprotein, VLDL: very low-density lipoprotein, CRP: C-reactive protein.

**Table 3:** Comparison of miRNA 423-5p between intervention and control groups.

Group	miRNA 423-5p		Mean Diff.	Paired ‘*t*’ Test *p* Value
	Pretest	Posttest		
**Intervention**	20.43 (±1.4)	25.5 (±1.66)	5.06	0.001
**Control**	25.35 (±1.6)	25.36 (±1.78)	0.015	0.917

**Table 4:** Correlation between miRNA 423-5p and BMI (kg/m^2^) differences.

Predictor for BMI (kg/m^2^) Difference	Correlation Coefficient “r”	B (95% C.I.)	*p* Value
miRNA 423-5p difference	−0.538	−0.3 (−0.48–−0.12)	0.002

**Table 5:** Comparison of miRNA 128-1 between intervention and control groups.

Group	miRNA 128-1		Mean Diff.	Paired ‘*t*’ Test *p* Value
	Pretest	Posttest		
**Intervention**	27.25 (±2.53)	24.53 (±2.53)	2.72	0.001
**Control**	20.69 (±1.43)	20.69 (±1.43)	0.005	0.989

**Table 6:** Correlation between miRNA 128-1 and BMI (kg/m^2^) differences.

Predictor for BMI (kg/m^2^) Difference	Correlation Coefficient “r”	B (95% C.I.)	*p* Value
miRNA128-1 difference	0.786	0.395 (0.28–0.51)	0.000

## Data Availability

No additional data beyond that published in this manuscript are available for sharing.
